# Enhanced hospital-based learning at a medical school through application of management principles – a case study

**DOI:** 10.1186/s12909-017-1024-y

**Published:** 2017-10-10

**Authors:** Anna Kiessling, Martin Roll, Peter Henriksson

**Affiliations:** Division of Cardiovascular Medicine, Department of Clinical Sciences, Danderyd University Hospital, Karolinska Institutet, Danderyd University Hospital, SE-182 88 Stockholm, Sweden

**Keywords:** Leadership, Undergraduate medical education, Clinical clerkship, Quality improvement

## Abstract

**Background:**

A hospital with all its brimming activity constitutes a unique learning environment for medical students. However, to organise high-quality education within this context is a task of great complexity. This paper describes a teaching hospital case, where management principles were applied to enhance the learning quality of medical education.

**Methods:**

Traditional attempts from the faculty had been unsuccessful in improving learning among medical students at a teaching hospital. We therefore applied management principles to be able to improve the learning quality. An evaluation was performed from the perspectives of management (course directors/ heads of health care departments), medical students, and physician supervisors. *Presages* were defined*,* including educational resources and management; *processes* were adjusted, including learning activities and staff schedules; and *products* were assessed.

**Results:**

Charting and benchmarking the use of local educational resources identified unused funding. Structured recurrent collaboration within resource utilization was established between course directors and heads of all concerned health care departments. By formulating a joint agreement, the identified assets were used to reorganise the course, to create constructive alignment, and to increase assigned supervisor time. This resulted in a sustainable improvement of learning quality and culture.

**Conclusion:**

By using management principles in combination with a scholarship of teaching and learning, it was possible to locate and redistribute educational resources in an effective way. This improved student learning and the learning culture of the health care departments. We propose that such an initiative could also be transferable to other contexts. Faculty leaders facing similar problems should consider the advantages of a structured collaboration with health care department heads.

## Background

The teaching hospital is a unique learning environment for medical students, with its pulse and activity 24 h a day, 7 days a week. However, clinical education does not have the highest priority, and several prerequisites have to be fulfilled to support high-quality learning in this type of context.

This case report assumes that becoming a professional is a social process that, at least partly, needs to take place in a context where the profession is practiced. The social interplay and learning processes at a health care workplace can be understood as a community of practice where members develop and uphold *a joint enterprise, mutual engagement,* and *a shared repertoire* [1]. According to Wenger, every community of practice has its specific leadership, mission, and culture defining its boundaries [2].

The *joint enterprise* of a hospital department is to provide high quality care of patients. To achieve this, systematic quality improvements are performed based on management principles and a systems approach to health care delivery, often adopted from engineering systems [3]. Further, cost containment has put a focus on cost effectiveness and quality of care. There are, in principle, two different economic steering systems: national health service systems funded by general taxation and social security health systems funded by earmarked premiums [4, 5]. Sweden, where the present study took place, has a national health service system. We believe that a focus on economics, irrespective of steering system, may cause a conflict between the somewhat competing missions of the teaching hospital: the care of patients and the provision of clinical education.

Deans of medical programmes at universities have a mission to improve health through education of novices in medicine to become competent and skilled physicians of the future health care system. Consequently, deans and course directors strive to promote overall quality of education. A promising survey among American hospital chief executive officers and medical school deans showed that a majority rated the alignment and relationship between themselves and their counterpart as “excellent” or “good” [6]. However, to our knowledge, operative alignment and relationship at department level are not that obvious, at least in Sweden. Course directors and heads of health care departments rarely systematically develop courses in collaboration.

The authors have observed that double chieftainships also complicate the fulfilment of high-quality clinical education. The chieftainship of education is separated from that of health care in terms of management methods, missions, and quality culture. Health care and higher education are regulated, at least in Sweden, by different laws and ordinances. Furthermore, the definition of teaching hospitals’ and universities’ shared responsibilities and accomplishments is often ambiguous for clinical education and research.

In Sweden, all higher health care education is funded by the government. The financial resources are made available at university level, which then redirects funds to the specific department responsible for the actual course. In parallel, the government provides all county councils with medical schools funding as a compensation for their participation in undergraduate medical education. These resources are subsequently made available to departments at the teaching hospitals based on the volume of students.

Approximately 280 medical students graduate each year from Karolinska Institutet, Stockholm, Sweden. In the clinical part of the programme, the university collaborates with Stockholm County Council, which offers health care services to approximately two million residents. Our experience is that unwanted effects may arise when educational funding is allocated at teaching hospital departments. Unspecific allocations of resources intended for clinical education can result in the funding disappearing into the total budget of the health care department.

To summarise, learning within a health care context is fundamental to becoming a physician. On the other hand, it is also the Achilles heel of a medical school. We planned this case study based on the assumption that the financial resources intended for supporting clinical education were not used in an optimal way at the department level of a teaching hospital and that a reorganisation was needed. The use of management principles in combination with academic leadership might be the possible strategy to apply.

The primary aim of this case study was to investigate if a sustainable joint collaboration between course directors and heads of health care departments was feasible. The secondary aim was to apply management principles to enhance the learning quality for medical students and to discuss the case from a theoretical perspective.

## Methods

### Study design

This case study describes an educational intervention and was performed with a before and after design, and a triangulated evaluation in three phases: *Presage, Process,* and *Product,* well known as the 3-P model [7].

### Setting

Danderyd University Hospital provides acute and elective care to 455,000 residents in Stockholm County, Sweden. It has 530 beds for in-patients and conducts a further 165,000 out-patient visits each year. There are 2800 employees, of whom approximately 390 are physicians, 1000 are nurses, and 660 are auxiliary nurses.

### Participants

*Course directors and heads of relevant health care departments:* During the study period, two course directors and three department heads were in charge.

*Students:* Each year 80 medical students participated in a transition to clerkship course followed by a clinical training period during two terms at the Department of Medicine and Cardiology at the hospital.

*Supervisors:* Each year 12 residents in internal medicine or cardiology participated as full-time supervisors for 4 weeks, training medical students in a transition to clerkship course.

### The evaluation process

The evaluation was structured based on answers to the questions suggested by D.A. Cook [8]: “Whose opinion matters?” and “What will really be meaningful to them?”

The first question: “Whose opinion matters?” was answered by the three participant perspectives: *The management perspective* (course directors and heads of relevant health care departments), *the student perspective*, and *the supervisor perspective*.

These perspectives were evaluated in three phases. In the *presages* phase*,* we defined key problems from all three perspectives. These key problems then formed the basis for phase two: the *process* to formulate an action plan and perform an intervention to reorganise the education at the department. The educational intervention is described in more details in the results section. Finally, the third phase, *product*, was formed by assessing the predefined outcome measures listed below.

### Outcome measures and data analysis

The outcome measures were assessed before and after the intervention and reorganisation of the course. The outcome measures were chosen based on Cook’s second question: ‘What will really be meaningful to them?’**.**

1. *Control of educational resources and costs and visualisation of the educational responsibilities of the department* were evaluated by comparing allocated educational resources at department level before and after. They were also evaluated by scrutinising organisational changes and new routines at the health care department level.
2. *Student learning* was evaluated using a questionnaire to students. The questionnaire included four items: the reception of students; views on supervision; goal achievement, and global impression of the learning environment. Students answered directly after the transition to clerkship course (*n* = 30–34 students per semester) in six consecutive semesters, i.e. before the reorganisation and the following five semesters. Questions were answered on a 10-grade Likert scale. Present perception was indicated by placing a X on the scale, a 10-cm line with verbal “anchors” expressing the extremes. The score of each item was obtained by measuring the centimetres from the left anchor to the X mark, with an accuracy of 0.5 cm. Mean and 95% confidence intervals were calculated.
3. *Working conditions and supervisor’s own learning* were evaluated by a questionnaire to supervisors and focus group interviews. A web-based questionnaire was sent to all physicians (*n* = 61) who had experience of at least one full-time period of 5 weeks as supervisors for students in the transition to clerkship course during a 5-year period after the intervention. The questions were answered on a 6-grade Likert scale with six fixed verbally anchored alternatives. Median and quartiles were calculated. Respondents also had the opportunity to provide free text comments. Focus group interviews with a purposive sample of these supervisors were conducted and analysed with qualitative content analysis as described in a previous paper [9].

## Results

Table 1 summarises the results of *presages, processes*, and *products* from the three perspectives.

**Table 1:** Presages, processes, and products from three stakeholders’ perspectives

Perspective	Presage	Process	Product
Management	Steering documents known and followed Financial educational resources allocated at hospital level and available at department level Charting of resources and costs at department level	Negotiate between course directors and heads of health care departments Visualise the educational processes Use all educational resources Establish a structure of collaborative recurrent follow-up	The managers’ collaboration to plan; perform; follow up quality, resources and costs; and improve educational outcome
Student	Examination results Student questionnaires and opinions Student volume	Establish a curriculum that supports student learning Create constructive alignment	The students’ experiences of their reception, their views of supervision, goal achievement, and global impression of the learning environment
Supervisor	Student schedules Scheduled time and logistics of supervision Course logistics	Establish continuity and competence among supervisors	The supervisors’ experiences of their own learning, benefits of being a supervisor, and satisfaction with management and organisation

### Presages before the educational intervention

Before the reorganisation of education, a situation with a suboptimal educational climate was observed. The two course directors identified a constant shortage of physician supervisors and a fragmentation and discontinuity of the supervisors’ working schedules. A mix of patient and supervisor work and a lack of resources made it impossible to align learning activities and to maximize the support of students’ learning.

There was no forum for information exchange and negotiation between course directors and the heads of the health care departments. There was neither follow-up nor transparency of educational resources and costs at department level. A range of external stakeholders at the hospital, university, and national levels had stated that it was impossible to get a clear view of the use of educational funding for the clinical parts of the medical undergraduate programmes. Educational funding for the salaries of supervisors and clinical teachers was also completely mixed with those resources aimed for the provision of health care at department level.

Student opinion at baseline verified the suboptimal educational climate [10]. Although the students passed their exams, they rated their satisfaction with the learning environment lower than nursing students at the same health care department, especially on the question regarding supervision. Student schedules were fragmented, and there was no student-supervisor continuity. At baseline, the supervisors were only scheduled part-time and had no allotted time for teaching. They had more or less concurrent stressful responsibilities as physicians. Selection as a supervisor for medical students was seen by residents as undesirable extra work.

### Process of the educational intervention

**The first step** was to identify the financial resources. Benchmarking at the three other teaching hospitals providing similar clinical courses for medical students showed that:

- The local structure and organisation of education differed substantially between the four teaching hospitals.
- The other teaching hospitals seemed to have more allocated time for supervisors involved in the course.
- All involved teaching hospitals had different routines to allocate and follow-up financial resources.
- Most of the identified teachers and supervisor positions were more or less mixed with patient and research work and thus had a combined financing.

The benchmarking showed substantial differences between the teaching hospitals concerning financial allocation of teaching resources and costs at department level. There was little collaboration between the teaching hospitals during the study period. This together with identified disparities in prerequisites and management made it impossible to perform a joint reorganisation at all hospitals. The course directors at the present hospital had tried all traditional approaches to quality improvement without effect. Based on their management backgrounds, they now decided to apply management techniques of benchmarking, charting, and analysis of budgetary allocation of resources and actual costs of education. An agreement was reached with the heads of the involved health care departments to perform a modelling of the present budget, costs, and outcomes and of a scenario with a future reorganised course.

This showed an imbalance between budget and costs for teaching and supervisor hours and identified an unused teaching asset of 1 million Swedish crones (SEK) per year (SEK 1 = Euro 0.11).

**The second step** was to reorganise the course to optimise support of student learning. A working group of physicians, teachers, supervisors, and staff was set up. The group was tasked to come up with a plan that optimised course schedules and logistics to achieve constructive alignment and an improved learning situation. The plan began prioritising the use of the identified resources to establish a structure with full-time supervisors during the important transition to clerkship course. The 5-week course was reorganised with three cornerstones:

1. Learning activities applying group-based student-activating learning techniques and the new schedule optimised time for interaction between students and supervisors during all learning activities.
2. Scheduled full-time supervisors which enabled recurrent individual feedback to students.
3. Groups of five to seven students were assigned a supervisor during thematically aligned sessions. The sessions were structured with problem-based group discussions, fundamental clinical skills training sessions, and patient meetings at the wards where students had sit-ins with their supervisor and learnt how to take a patient history and how to examine patients for the first time.

The reorganisation of the course also aimed to provide continuity and competence among supervisors. The physicians, mostly internal medicine attending physicians, were scheduled 5 weeks as supervisors, allowing time for own basic training in pedagogy, including problem-based tutorship, self and collegial critical reflection regarding student learning, how to educate in the clinical setting, and how to give constructive feedback. These learning outcomes gained from serving as a supervisor in this setting were visualised in dialogues with the heads of the health care departments. These heads realised that to be a supervisor could help residents to achieve some of the intended learning outcomes stated in the attending physicians’ residency training requirements.

### Product of the intervention – Outcomes from three perspectives

#### Management perspective

It is now established routine to have regular meetings between heads of the clinical health care department and the course directors, including quarterly follow-up, on the use of educational resources at the department. Discussions resulted in visualising silent problems (see below) and a common view of the educational commission. An example is that colleagues now are visualised as appointed supervisors, not as being on leave, in the weekly physician schedule. To date, more than 100 of the residents at the involved clinical departments have performed at least one 5-week period as supervisor on the course. The competences they achieve from this experience are now recognised in their residency-learning portfolio. Most of them still work at the same health care department. The growing number of residents and consultants with this experience at the concerned health care departments appears to have changed the collegial culture to make it a more education-friendly climate. Over time, a shared educational view has developed. Medical students are now recognised as future colleagues and the supervisors as their important educators. In addition, students now nominate “The best supervisor of the year” and the prize-winner is recognised at a meeting for all physicians at the departments.

#### Students and supervisor perspectives

**Fig. 1:**
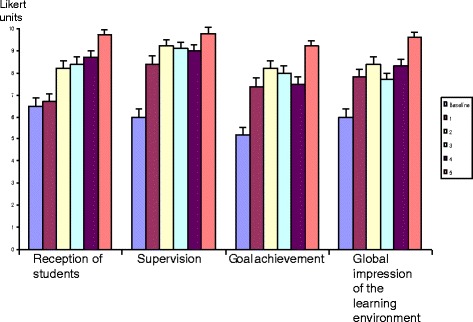
Medical students’ perceptions of the transition to clerkship course. Answers were given on a 10-grade Likert scale with verbal anchors expressing the extremes. The first bar labelled [Baseline] denotes answers from students participating in the course before the reorganisation. Bars labelled [1–5] denote answers from students participating in the course during the following five semesters, after reorganisation of the course. Mean and 95% confidence intervals are given (*n* = 30–34 each semester; response rate > 85%)

**Fig. 2:**
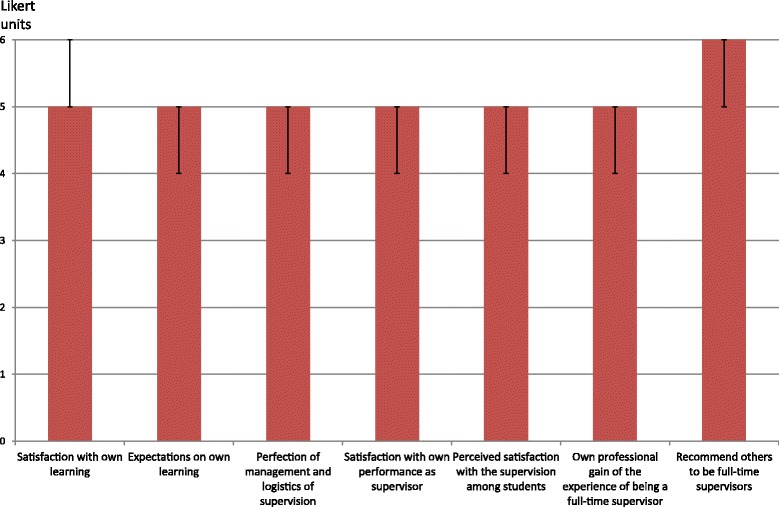
Physician supervisors’ perceptions of being a full-time supervisor in the reorganised course. Answers were given on a 6-grade Likert scale with six fixed verbally anchored alternatives. Median and range are given (*n* = 61)

The reorganisation resulted in mutually perceived satisfaction and professional learning among both students and supervisors. Figure 1 shows that the ratings of students increased significantly from a span of five to six on a 10-point Likert scale before the change to well above nine after reorganisation of the course design. An associated very high satisfaction with the new organisation was also found among supervisors, as shown in Fig. 2. The following comments illustrate the residents own benefits of being a supervisor in the reorganised course.

*“The interaction between me, the students, and the patients has given me self-confidence in other assignments as supervisor. It (to be supervisor in this course) was an eye opener for my interest in teaching that has remained since then.”*

*“I have increased my awareness of my different roles: as physician, as supervisor, as team leader, etc.”*

*“I have developed my skills in explaining and answering questions.”*

*“To be a supervisor has improved my physical exam skills, both to perform and to demonstrate them in a structured manner.”*

In a previous publication [9], we have presented how supervisors in this course perceived their role based on data from focus group interviews. Four themes emerged: being present in the moment; being a catalyst for learning; being an expert; and supporting students’ sense of coherence. We concluded that full-time as supervisor allowed physicians to pay close attention to the student’s learning process and interact both individually and with groups of students. Their experiences and expertise were used to facilitate students in their own learning and to give qualified feedback. The supervisor continuity and presence created a coherent learning environment for students to realise the true meaning of being a doctor.

## Discussion

This case study started with a suboptimal educational situation with reduced resources and quality that necessitated a new approach. We had to think outside the “educational university box” and use additional perspectives and skills to those normally associated with curriculum development. We applied management principles and a joint leadership perspective. Further, we negotiated on feasible and successful solutions based on benchmarking, cost charting, and consensus discussions. The results were mutual benefits, within budget limitations, in learning quality for both students and supervisors at resident level. Our finding that the allocation of intended resources improved workplace learning is in line with the results of O’Brien and Poncellet [11], which showed that medical schools in the United States and Canada that funded transition to clerkship courses provided more time for clinical immersion than those without funding.

### Visualisation of a silent problem

This case is an example of visualisation of a silent problem. Managerial responsibilities and financial control are crucial to quality in both health care and higher education. However, applying the terminology of Souba et al. [12], we state that educational leaders’ lack of sole financial control and managerial responsibility for education performed in the health care environment is an elephant of great importance when it comes to educational development. *“Elephants’ are ‘obvious problems that impair performance but which the community collectively does not discuss or confront”.* According to Souba this collective avoidance is called organisational silence. An example of this silence in our case was the response we received when we approached course directors at the surrounding teaching hospitals in an attempt to try to engage them in solving the financial problems. We were met with silence and with such arguments as that this problem was impossible to solve because the heads of the health care department owned it and that this could not be changed.

### Scholarship of teaching and learning: an academic perspective on leadership of health care education

Almost all teachers at medical schools and a lot of the heads of health care departments at teaching hospitals have academic degrees and have completed a research education. This implies that they are well trained to perform scholarly work and academic leadership. However, scholarly research and teaching principles do not automatically translate into high quality management performance or vice versa. In this case, we learnt that recurrent collaborative dialogues and negotiation between responsible chairs of both organisations facilitated this translation. Trigwell et al. [13] presented a multidimensional model describing the scholarship of teaching, including information, reflection, communication, and conception. In Table 2, we applied this model to the experience of our case and formulated an interpretation of the scholarship of teaching as an academic perspective on management of education at health care department level. Furthermore, our scholarly emphasis on a transparent notification of experiences, results, and reflections on outcome is consistent with contemporary views of the very nature of effective professional and organisational learning [14, 15].

**Table 2:** Principles of scholarship of teaching and learning on department level at a teaching hospital

	Information	Reflection	Communication	Conception
Clinical department chairs	Being informed on pedagogic techniques that facilitate workplace learning	Why conducting education at this department? How do the students’ results impact the health care today and tomorrow?	Include education results in follow-up reports and management discussions Negotiate with course directors	See clinical teaching as capacity building and as an activity promoting health care quality See student learning as a guarantor of future competence
Course directors at medical school	Being informed on laws and regulations of resource allocation and actual educational costs	Who are the consumers of the results of education? What really matters to them? How can we use the available resources to maximise learning outcome?	Report own results at local and national meetings Negotiate with clinical department chairs	See clinical teaching and learning as an essential activity of high quality health care See student–patient meetings as the outpost of student learning

This multi-dimensional model of scholarship of teaching and learning is modified and adapted from Trigwell et al. [13]

### Change in the social learning system

Another important mechanism supporting the success in this case was the change that took place, to our understanding, in the concerned communities of practice [1]. In later work, Wenger [2] defines three forms of participation or belonging to a community of practice – and to a social learning system: *engagement*, i.e., doing things together, talking, producing artefacts; *imagination*, i.e., constructing an image of ourselves, of the community, and of the world to orient ourselves, to reflect, or to explore possibilities; and *alignment*, i.e., making sure that the local activities are sufficiently aligned with other processes so that they can be effective beyond our own engagement. This case study shows a drift from nonparticipation to participation, from two separate communities of practice to a social learning system with shared engagement in the students’ and the residents’ learning processes, a shared “mental model” of the aims and incitement of these processes at the health care department, and an alignment that visualises the benefits for patient care by student and supervisor participation in the health care community. Even if participation in the community of practice can be hypothesised to support learning, the students’ or even the physician supervisors’ belonging to the community of practice at a ward cannot be taken for granted. It has to be earned and supported by the social system at the individual, community, and management levels.

According to the theories presented by Wenger [2], we used the core processes and the boundaries between the communities of practice as bridges for the development of learning opportunities. We aimed to visualise a need to learn and to promote growth of a shared social learning system and an awareness that health care and faculty leaders could influence this system but neither control nor own it.

## Conclusion

Our conclusion is to emphasise the importance of thinking outside the university box when reorganising clinical courses. In this particular context, we used a triangulated approach and structured a joint collaboration and negotiation between local leaders of university and health care departments to improve clinical learning. This approach made it possible to earmark all educational resources and to use them to implement and sustain a clinical hospital-based education of high quality standards. Furthermore, we propose that the combined use of the scholarly principles of leadership of teaching and learning and of health care respectively has a potential to improve both educational quality and working conditions of health care. It has a potential to facilitate development of a social learning system and a workplace culture emphasising mutual learning. We recommend that others facing similar educational problems consider the potential of a joint scholarly leadership approach and a structured collaboration with health care department heads.
